# Supplementary material for: Biomaterials Based on Carbon Nanotube Nanocomposites of Poly(styrene-b-isobutylene-b-styrene): The Effect of Nanotube Content on the Mechanical Properties, Biocompatibility and Hemocompatibility
Source: Nanomaterials (Basel). 2022 Feb 22;12(5):733. doi: 10.3390/nano12050733 (PMC8911977; doi:10.3390/nano12050733)
Supplement: Supplementary file 1 [file nanomaterials-12-00733-s001.zip › nanomaterials-1585970-supplementary.pdf]

nanomaterials

Supplementary Material

Biomaterials Based on Carbon Nanotube Nanocomposites of Poly(styrene-*b*-isobutylene-*b*-styrene): The Effect of Nanotube Content on the Mechanical Properties, Biocompatibility and Hemocompatibility

Maria A. Rezvova [1,*], Pavel A. Nikishau [2], Miraslau I. Makarevich [2,3], Tatiana V. Glushkova [1], Kirill Yu. Klyshnikov [1], Tatiana N. Akentieva [1], Olga S. Efimova [4], Andrey P. Nikitin [4], Valentina Yu. Malysheva [4], Vera G. Matveeva [1], Evgeniia A. Senokosova [1], Mariam Yu. Khanova [1], Viacheslav V. Danilov [5], Dmitry M. Russakov [6], Zinfer R. Ismagilov [4], Sergei V. Kostjuk [2,3,7] and Evgeny A. Ovcharenko [1]

[1] Department of experimental medicine, Research Institute for Complex Issues of Cardiovascular Diseases, 650002 Kemerovo, Russia; bio.tvg@mail.ru (T.V.G.); klyshnikovk@gmail.com (K.Y.K.); t.akentyeva@mail.ru (T.N.A.); matveeva_vg@mail.ru (V.G.M.); sergeewa.ew@yandex.ru (E.A.S.); khanovam@gmail.com (M.Y.K.); ov.eugene@gmail.com (E.A.O.)
[2] Research Institute for Physical Chemical Problems, Belarusian State University, 220006 Minsk, Belarus; nikishau@bsu.by (P.A.N.); miraslau.makarevich@gmail.com (M.I.M.); kostjuks@bsu.by (S.V.K.)
[3] Department of Chemistry, Belarusian State University, 220006 Minsk, Belarus
[4] Institute of coal chemistry and material science, Federal Research Center of Coal and Coal Chemistry SB RAS, 650000 Kemerovo, Russia; efimovaos@mail.ru (O.S.E.); nikitinandreyp@yandex.ru (A.P.N.); v23091@yandex.ru (V.Y.M.); zinfer1@mail.ru (Z.R.I.)
[5] Research Laboratory for Processing and Analysis of Big Data, Tomsk Polytechnic University, 634050 Tomsk, Russia; viacheslav.v.danilov@gmail.com
[6] Institute of fundamental Sciences, Kemerovo State University, 650000 Kemerovo, Russia; dm.russakov@gmail.com
[7] Institute for regenerative medicine, Sechenov First Moscow State Medical University, 119991 Moscow, Russia
* Correspondence: rezvovama@gmail.com

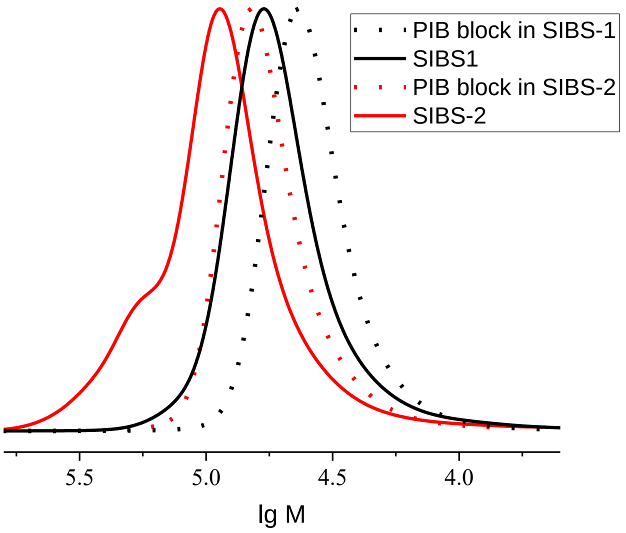

Figure S1. SEC traces of polyisobutylenes and tri-block copolymers. See Table 1 for the details on the properties of SIBS-1 and SIBS-2.

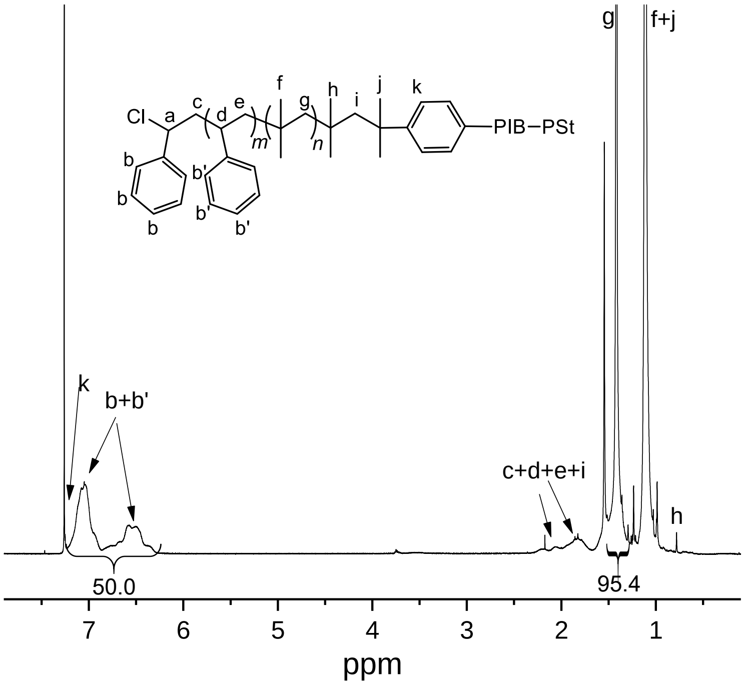

Figure S2. ^{1}H NMR spectra of the SIBS-2. Signal a was not detected due to high molecular weight of triblock copolymer sample.

$$\omega_{St} = \frac{I_{b+b'} \times 104/5}{I_{b+b'} \times 104/5 + I_g \times 56.1/2} \times 100\% \quad \text{(S1)}$$

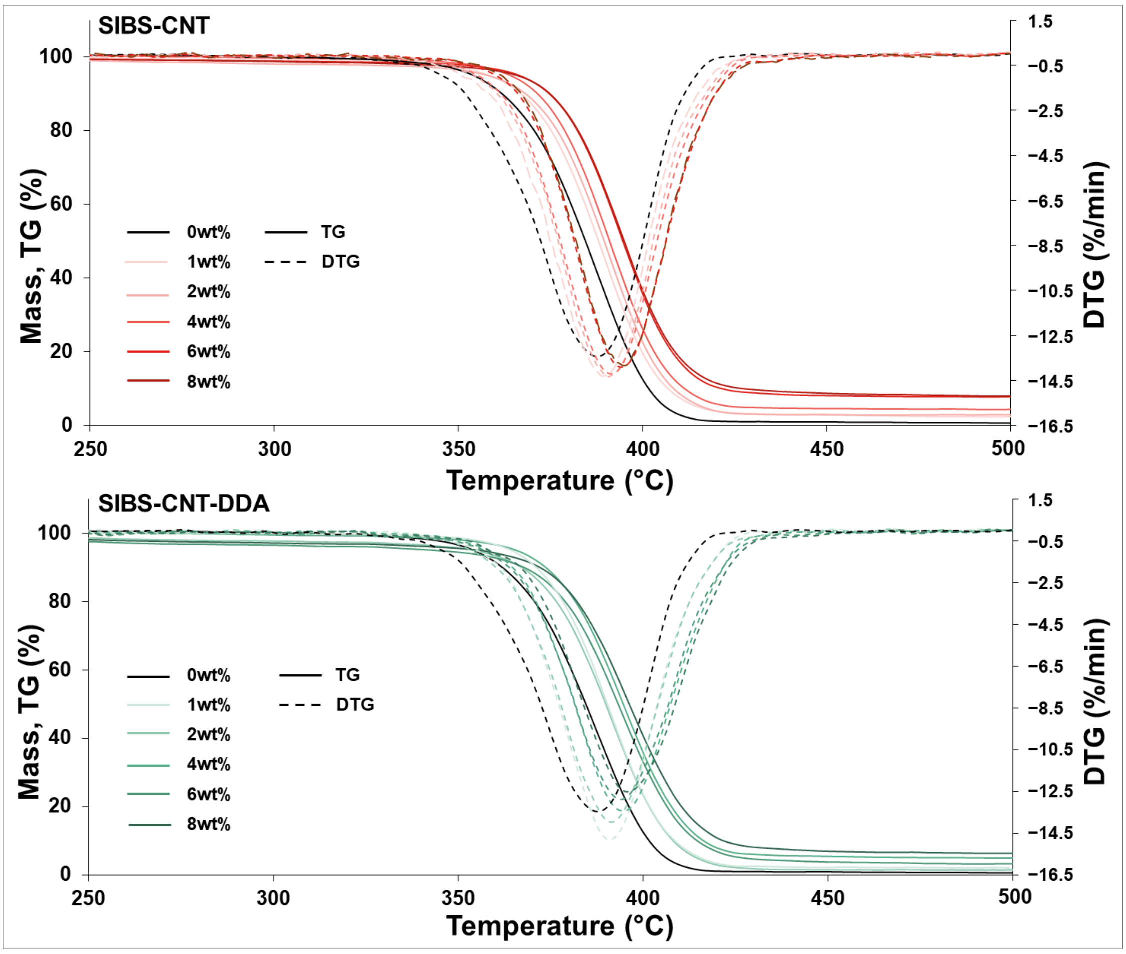

Figure S3. TG and DTG curves of nanocomposite films.

Table S1. The results of determining the particle size of CNT dispersions in chloroform by the DLS method.

Sample	Particle size, nm
CNT	0.96 ± 0.15
	11.70 ± 1.02
	34.03 ± 5.13
	141.3 ± 29.48
	654.5 ± 130.1
CNT-COOH	1.50 ± 0.15
	11.70 ± 2.03
	68.06 ± 13.03
	342.0 ± 71.91
CNT-DDA	1.29 ± 0.19
	18.17 ± 0.99
	55.48 ± 8.90
	115.9 ± 21.85
	499.4 ± 93.73

Table S2. Decomposition parameters of composite films and pure polymer.

Sample	T_{onset} (°C)	T_{max} (°C)	T_{end} (°C)	Residual weight, %
SIBS	365.9	387.6	402.8	0.24
SIBS-CNT-1%	370.8	389.6	404.7	1.77
SIBS-CNT-2%	372.9	389.7	406.2	2.14
SIBS-CNT-4%	374.1	391.2	406.6	3.39
SIBS-CNT-6%	377.2	394.5	409.8	7.06
SIBS-CNT-8%	377.5	395.2	410.0	7.22
SIBS-CNT-DDA-1%	373.6	390.9	406.2	2.23
SIBS-CNT-DDA-2%	373.1	391.2	407.7	1.50
SIBS-CNT-DDA-4%	376.7	395.0	410.8	3.84
SIBS-CNT-DDA-6%	375.8	393.9	410.9	3.34
SIBS-CNT-DDA-8%	377.7	395.7	413.3	6.35
